# Heterogeneity and agency in the contemporary food regime in Switzerland: among the food from nowhere, somewhere, and here sub-regimes

**DOI:** 10.1007/s41130-024-00207-y

**Published:** 2024-03-20

**Authors:** Rike Stotten

**Affiliations:** Department of Sociology, University of Innsbruck, Universitätsstrasse 15, 6020 Innsbruck, Austria

**Keywords:** Food regime theory, Alternative food system, Corporate food regime, Socially green capitalism

## Abstract

This study empirically illuminates the contemporary food regime in Switzerland to understand the organisation of food production, distribution, and consumption. From the perspective of food regime theory, it highlights in detail the (inter)relationships in the food regime between the food from nowhere, somewhere, and here sub-regimes using empirical means. Heterogeneous structures, processes, and relations that coexist within an umbrella food regime are examined. To address the criticisms of food regime theory ignoring social agency, this study further reveals collective agency and addresses the role of alternative food systems within the food regime in Switzerland. In-depth document analysis and subsequent qualitative data collection relying on expert interviews were performed. This study illustrates the collective agency shaping the contemporary food regime in Switzerland, encompassing private companies, relevant media, as well as associations and unions involved in farming, processing, and consumption. These influential entities and actor-networks advance different sub-regimes of food from nowhere, somewhere, and here that reflect the heterogeneity of the contemporary food regime in Switzerland. However, the data did not provide sufficient information to determine the collective agency of actors within the alternative food system. The dynamics of the food regime are shaped by contested social practices, which are influenced and interpreted through social agency. This results in an overlap of the sub-regimes that has led to strong counter-movements within the contemporary food regime in Switzerland.

## Introduction

The ‘multiple crises of capitalism’ (e.g. Clapp & Moseley, 2020; Gliessman, 2022), expressed in economic and political instability, climate change, environmental degradation, and social inequality (Rosol, 2020) bring forward the current problems of the contemporary food system shaped by corporate power. Simultaneously, so-called alternative food systems (AFS) oppose the corporate power in the food regime (Plank et al., 2020). Studies have proven this substantial move for re-localisation as a reaction to tendencies of globalisation, commodification, and de-localisation (e.g. Schermer, 2015). Within the framework of food regime theory, this move has been discussed in terms of social movements and their struggles connected to food sovereignty (Alonso-Fradejas et al., 2015; Bernstein, 2016).

These food regimes are conceptualised as ‘relatively bounded historical periods in which convergent expectations govern the behavior of farmers, firms, and workers engaged in all aspects of food growing, manufacturing, services, distribution, and sales, as well as government agencies, citizens and consumers’ (Friedmann 2004: 125). This homogenising food regime perspective serves as an organising concept for the interpretation of food systems under capitalism. However, it marginalises a large arena of food production and consumption beyond the dynamics of the global food system (Friedmann, 2005; McMichael, 2000b, 2009), as not all food production and consumption conform to a macro-level pattern. In contrast, these small- and mid-scale food systems have often proved to be socially and ecologically embedded (Campbell, 2009), underscoring the significance of social relations within the food system. Therefore, in debates on the so-called third or corporate food regime, different simultaneous phenomena emerged conceptually: the ‘food from nowhere’ sub-regime shaped by neoliberalism (McMichael, 2009), the ‘food from somewhere’ sub-regime characterised by denser ecological feedback (Campbell, 2009), and the ‘food from here’ sub-regime reliant upon localism (Schermer, 2015).

As the food regime theory perspective neglects heterogeneous structures as well as agency, this study aims to better understand these aspects. Therefore, this study sheds light on the contemporary food regime in Switzerland through the elaborated lens of food regime theory and reveals the heterogeneous structures and agency within it. Furthermore, it reflects on the underexplored interrelationships among the food from nowhere, somewhere, and here sub-regimes, and the aspirations of corporate adoption of the food from here sub-regime.

## Conceptual lens: the food regime theory

A food regime is a ‘rule-governed structure of production and consumption on a world scale’ (Friedmann, 1993, pp. 30–31). Thus, the food regime theory (FRT) is an approach to understanding the global organisation of food production, distribution, and consumption in terms of political economy (Friedmann, 1995). It investigates how food chains enmesh and transform different cultural areas through globalisation and commodification, and its analysis functions as a critique of food systems in a capitalist world (Bernstein, 2016). With its analysis, FRT reveals lock-ins and path dependencies that have led to relative stability over several decades (Langthaler et al., 2023). The underlying assumption is that forces in different periods have always shaped food regimes. The UK-centred global food regime emerged first, relying on cheap food and raw material from the colonies that enabled European industrialisation. This food regime was followed by the US-centred regime after World War II that reversed the flow of food from the Global South to the Global North and was shaped by the global spread of industrial agriculture through the ‘Green Revolution’. This food regime was gradually replaced by the third food regime dominated by the World Trade Organization (WTO) and the rise of corporate power (McMichael, 2013).

### The (third) corporate food regime

The third food regime is shaped by a set of rules institutionalising corporate power in the global food system. The WTO is a leading institution whose values are exercised through several trade agreements, such as the North American Free Trade Agreement (NAFTA) (McMichael, 2009). In his elaborations, McMichael highlights four key features of the third food regime (Bernstein, 2016). First, the neoliberal dynamics of market liberalisation, the privatisation of previously public services, and the corporate-friendly regulations of intellectual property rights guide developments in the regime (Otero, 2016; Pechlaner & Otero, 2010). Second, the regime is based on accumulation by dispossession—the displacement of farming cultures through low agricultural prices, conversion of land for agricultural export, and intertwining of state policies and corporations’ economic interests (McMichael, 2009). Third, it relies on ecologically destructive, increasingly industrialised agricultural production that subverts social contexts (Bernstein, 2016; McMichael, 2009); and fourth, it is shaped by a delocalisation of production and consumption (McMichael, 2009). Friedmann (2005, 2016) outlines the emergence of a ‘green capitalism’ that seeks to adopt social movements’ claims regarding fair trade, health, sustainability, and animal welfare. In contrast to McMichael, she (Friedmann, 2005, 2016) perceives the influence of the global environmental movement and the adaptability of capitalist companies as impactful. Whereas McMichael (2009) considers this ‘corporate’ food regime as consolidated, Friedmann (2005) presents it as the emerging ‘corporate-environmental’ food regime (see also Bernstein, 2016). With its divergent foci, they nevertheless draw distinct but not necessarily contradictory concepts defining a food regime.^1^

However, this debated dominant food regime of accumulation addresses tendencies such as the growth of transnational corporate power, new regulatory frameworks (e.g., CAP), the intensification of production, greater flexibility and specialisation of the food system, the financialisation of food systems, global and direct sourcing, new production-consumption relationships, increased demand for healthy and functional foods, and critiques concerning the ecological sustainability of food systems (McMichael, 2009; Smith et al., 2010). These divergent aspects have resulted in a tension that shapes this food regime, which ‘embodies a central contradiction between a “world agriculture” (food from nowhere) and a place-based form of agro-ecology (food from somewhere)’ (McMichael, 2009, p. 147). Debates on these tensions brought forward conceptualisation what can be understood as sub-regimes within this dominant food regime. The food from nowhere sub-regime is characterised by the severe liberalisation and commoditisation of supply chains under corporate control: the harmonisation of production standards, substitutability along the supply chain, national food regulation limitation, and loss of food’s cultural meaning. Relying on the effects of the 2008 financial crisis, finance capital underwrites the corporate control of land and resources overseas by companies in the agri-food chain (Burch & Lawrence, 2009), shaping the food from nowhere sub-regime. These companies, ‘Big Food’ corporations (Clapp & Scrinis, 2017), dominate the packaged food industry, including brands such as *Nestlé*, *Kraft*, and *General Mills*. This financialisation further drives digitalisation expressed as biotechnology (e.g. genome editing), industry 4.0 (e.g. automation, robotics, artificial intelligence, digitalisation in the food processing, and packaging sector), or smart farming (farming technologies). This digital turn favours the food from nowhere sub-regime as digital features demand massive financial investments mostly implemented by large-scale agriculture, processors, or retailers (Prause et al., 2021).

The food from nowhere sub-regime faces resistance from social movements, exemplified by La Via Campesina (McMichael, 2000a), which advocates for transformative change. Following this, Campbell (2009) speaks of a food from somewhere sub-regime—in opposition to the nowhere sub-regime—where food is rooted in culture and consumers value its social and ecological embeddedness. With the establishment of global food audit initiatives (Campbell, 2009), retailers selling food from somewhere have commandeered this strategy for customer acquisition and loyalty. For example, adopting carbon footprints and organic criteria allows consumers to consider ethical issues, such as food’s ecological feedback and fair trade. Moreover, the WTO supports this strategy institutionally by presenting Geographical Indications within their Agreement on Agriculture (Friedmann & McNair, 2008). The emergence of the food from somewhere sub-regime was further driven by several food scandals, such as mad cow disease and *Escherichia coli* outbreaks, which led to a cultural delegitimation of the food from nowhere (Campbell, 2009). A summarisation of the food from somewhere sub-regime reveals similarities to Friedmann’s (2005, 2016) perspective on the emerging environmental-corporate food regime. However, debates have emerged regarding green capitalism’s contribution towards sustainability (Campbell, 2009; Guthman, 2014).

Regarding the food from nowhere and somewhere sub-regimes, Schermer (2015) considered the de-commodification of food and developed a food from here sub-regime, in view of the substantial re-localisation movement that has produced new forms of agency within food chains. Driven by rising concerns about the environment and the social effects of agriculture and food production globalisation, support for alternative food systems^2^ (AFS) has increased (Rosol, 2020). These comprise alternative food (e.g. organic, vegan, local food labels), alternative food networks (e.g. direct marketing, community-supported agriculture, fair trade), or alternative economies (e.g. food sharing, solidarity economy, social enterprises). Central to the last two aspects is the fact that such approaches ignore the traditional binary distinction of active producers and passive consumers, striving for cooperation that confronts the conventional mainstream food system (Schermer, 2015). Both approaches question the standardised and commodified mode of food supply (Renting et al., 2003) and seek local, healthy, and transparent supply networks (Smith et al., 2010). Corporations and financial investors have long ridiculed this trend; however, today, the conventionalisation of alternative food as food from somewhere, such as organic or vegan food, has been implemented by many retailers, processors, and farms (Rosol, 2020). So far, neither alternative food networks nor alternative economies have been adopted by the food from nowhere or somewhere sub-regimes as de-commodification presents substantial barriers to overcome. However, several FR actors see the potential for profit from alternative food networks and alternative economies (Rosol & Barbosa, 2021).

### Empirical investigations of food regimes

Food regime analysis has been empirically applied to reveal the first and second food regimes along ‘fundamental questions in the changing political economy (‘transformations’) of capitalism since the 1870s’ (Bernstein, 2016: 613). Although FRT itself does not provide a heuristic model for empirical analysis, a systematic overview of the food regime characteristics was provided by Bernstein (2016, p. 614) who proposed the following key elements: international state system; international divisions of labour and patterns of trade; ‘rules’ and discursive (ideological) legitimations of different food regimes; relations between agriculture and industry, including technical and environmental; change in farming; dominant forms of capital and their modalities of accumulation; social forces (other than capitals and states); tensions and contradictions of specific food regimes; and transitions between food regimes. For the third food regime, he additionally identified the key element ‘alternatives’.

Besides referring historically to the first and second food regimes, several attempts have been made to investigate contemporary regional and national dominant food regimes using multi-actor and multi-scale approaches (e.g. Escher, 2021; Green, 2022; Jakobsen, 2019; Otero, 2012; Pechlaner & Otero, 2010; Schermer, 2015; Wang, 2018; Werner, 2021; Wilson, 2016). Scholars have focused predominantly on the Global South, including India (Jakobsen, 2019), the Caribbean (Wilson, 2016), the Dominican Republic (Werner, 2021), and Argentina (Lapegna, 2016), to analyse, for example, labour as an expression of capital (Jakobsen, 2021), the state as an outcome of uneven regulatory development (Werner, 2021), or changing producer–consumer relations (Schermer, 2015). Efforts to investigate food regimes considering FRT in the Global North are limited so far (see, for an exception, Schermer, 2015).

### Critics of food regime theory

Despite its merits, FRT suffers limitations of which two will be discussed below and addressed in this study. First, a simplifying view that tends to overemphasise homogeneity within each food regime; and second a lack of attention to agency. Addressing the first, FRT particular strength lies in examining the stabilising dimensions of the respective food regime that results in an overemphasis on stability and coherence. Further, it does not take into account the heterogeneous structures of the FR and the interrelationships of sub-regimes that participate in an umbrella regime and thus ignores its heterogeneity (Langthaler et al., 2023). Whereas FRT neglects periods of transition between regimes in its initial ideas (Friedmann & McMichael, 1989), Friedmann (2016) recently stated ‘that food regime analysis is most useful today as part of a wider set of analyses of transitions’ (672) and suggested to ‘widen the conversation’ of FRT by enriching it with other theoretical approaches to examine social change. Nevertheless, so far, even the ‘contours of the third food regime remain undefined’ (Werner, 2021, p. 1).

The second criticism results from an undertheorised conceptualisation of the relationship between capitalism and the modern state that does not consider agency and social relations (Otero, 2016; Tilzey, 2019). Additionally, Rioux (2018) argues that FRT’s broad perspective conceals the impact of sub-national processes within the food system (see also Moran et al., 1996; Pritchard, 1996) and highlights the power of local communities—shaped by agency and values beyond the economy—such as farming cooperatives that influence the regional appearance of agro-commodity chains. McMichael (2016) attributes agency most prominently to corporations in the third food regime, but neglected the role of consumers and consumption. Also the role of social agency in family farms is underestimated, where ‘a degree of autonomy (agency) in deciding what to produce and how to market their output’ is retained within the respective agricultural policy setting (Atkins & Bowler, 2003, p. 33). More precisely, studies on FRT mostly ‘downplay or ignore local agency’ (Pechlaner & Otero, 2010, p. 181). McMichael (2009) also stresses the limited understanding of AFS, such as small- and middle-scale initiatives (Plank et al., 2020), in the food regime perspective because it discounts the social reproduction of alternative food cultures that rely on ecologically sustainable practices beyond economic rationality.

### Aim of the study

Friedmann (2005, 2016) highlights the third food regime as elusive and food systems as complex. Thus, empirical analysis is limited on selected substantial components (Bernstein, 2016), which also comes to bear in this study. Furthermore, the global long-term perspective of the FRT has been criticised as difficult to grasp for empirical analysis. Therefore, this study, as one puzzle piece in the global perspective, illuminates empirically the contemporary (third) food regime in Switzerland to understand the organisation of food production, distribution, and consumption. It highlights in detail the (inter)relationships between the food from nowhere, somewhere, and here sub-regimes using empirical means. Heterogeneous structures, processes, and relations that coexist within an umbrella food regime are brought to fore, presenting a perspective that the globally focused FRT tends to neglect. To address also the criticisms aimed at ignoring social agency, this study further reveals collective agency, and addresses the role of AFS and consumption within the food regime in Switzerland.

While the ‘methodological nationalism’ critique within the FRT has been raised (Lapegna, 2016), I acknowledge Switzerland as a relatively small country, where regions frequently align with cantons, showcasing significant autonomy yet interconnectedness at the state level. However, the nation’s size imposes limitations on comprehending the global integration of the food regime, necessitating further research.

### Sharpening the theoretical lens

For the empirical analysis, the theoretical lens on agency will widen the food regime perspective. The origin of the term ‘agency’ refers back to its Latin roots *agentia*, meaning *doing.* Within the plethora of social research, scholars (e.g. Burkitt, 2016; Hitlin & Elder, 2007) define agency regarding their specific interest of research which results in a diversity of theoretical approaches to agency. Agency in this study is conceptualised as ‘the capacity of human beings to shape the circumstances in which they live’ (Emirbayer and Mische, 1998, p. 965) emphasising the agentic dimension of social action. It encompasses the ability of actants, whether individuals or social groups, to not only take deliberate actions but also analyse the outcomes of their actions. Deriving from Latour (2005) who does not only ascribe agency to human beings but also to other actants (e.g. animals), actor-network theory, for example, ascribes the ability to act to actor-networks rather than in individuals (Callon & Muniesa, 2005). Therefore, for this research agency is considered ‘a collective, hybrid phenomenon resulting from the associations that are established among human, material, and natural entities.’ (Le Velly & Dufeu, 2016, 175). Referring to agency as a collective phenomenon integrates a relational sociology perspective. This angle highlights the relational connections among the so-called interactants that appear as networks of relations (actor-networks). As such, they produce interdependencies and joint actions that produce a particular effect, even if the outcomes are unintended (Burkitt, 2016). Within this relational perspective, one party of collective agency, mostly unconsciously, acts on behalf of another (Shapiro, 2005). To understand agency within the contemporary food regime, this study looks on collective agency of actor-networks in a relational perspective.

## Materials and methods: empirical approach to investigating the contemporary food regime in Switzerland

### Methodological approach

To empirically investigate the contemporary food regime in Switzerland, a two-step methodological approach was applied. Employing method triangulation, the outcomes of the document analysis and qualitative interviews contributed to the development of a thorough understanding of the phenomena (Bowen, 2009).

First, a document analysis (Bowen, 2009) was conducted to obtain insights into the food regime in Switzerland. Documents such as research reports, scientific publications, public government documents, official statistics, annual reports, articles of association, and websites of organisations of interest were analysed and compiled in a case study report^3^ according to defined categories that broadly shape the national context of the food system: agricultural structure, agricultural policy, agricultural actors, trade and retailing, consumption, and alternative food systems.

Second, a qualitative research extracted different perceptions and dimensions to reconstruct the food regime and especially to reveal inherent conflicts, cooperation, and multi-scalar linkage in Switzerland.^4^ Expert interviews (Bogner et al., 2009) were conducted between October, 2021, and January, 2022. Twelve experts were selected per a defined sampling strategy and represented one of the following social groups: 1. state: political representatives of parties, interest groups; 2. important political-economic actors: financial actors, biotech actors; 3. civil society: representatives of (agrarian, food, environmental) social movements and NGOs; and 4. actors in the production of knowledge: agrarian experts and researchers (see Table 1). Due to COVID-19 restrictions, interviews were conducted online (bigbluebutton) and lasted between 45 and 90 min. All the study participants provided written informed consent, and the study design followed the ethical rules and considerations reflected in the European Commission document “Ethics for researchers” (European Commission, 2013) and the “European Code of Conduct for Research Integrity” (ALLEA, 2017). The interview guideline served to generate narratives to exploratively explore the context of the contemporary food regime, its actors and power relations, and conflict and cooperation from the perspective of the experts.

**Table 1:** List of experts

Expert	Category	Actor
1	Agrarian experts, researchers	Research
2	Agrarian experts, researchers	Research and education
3	Agrarian experts, researchers	Research and education
4	Civil society	Social movement, peasant farming
5	State in a narrow sense and important political-economic actor	Agriculture/farming and politics administration
6	Important political-economic actor	Food business and trade
7	Important political-economic actor	Agro-input (seed, chemicals)
8	State in a narrow sense	Politics/administration
9	Important political-economic actor	Environment/agro-ecology
10	Important political-economic actor	Agro-input (seed, chemicals)
11	Important political-economic actor	Food business and trade
12	Civil society	Environment/agro-ecology

Anonymised transcripts were descriptively evaluated using qualitative content analysis following Mayring (2019). This systematic, rule-guided, and theoretically grounded approach is based on the inductive development of codes and the application of deductive verification of the codes to the research questions. Forty inductive codes were developed and deductively clustered into seven categories created in the broader project setting to investigate conflicts, cooperation, and multi-scalar interplays in food regimes in different national contexts (Plank et al. (n.d.)). The three categories, agency, alternative food systems and sub-regimes, were analysed for the aim of this paper.

### Context of the Switzerland case study

With a surface of 41,285 km^2^ and a population of 8.7 mio (2021), Switzerland is a densely populated but small country based on a direct democratic system and located in central Europe. As its dominant food regime is strongly enmeshed (among others) with its agricultural structure, policy, trade and retail, consumption, and alternative food systems, the country’s key characteristics will be presented here.

#### Agricultural structure

Swiss agriculture has undergone a structural transformation over the past few decades; the number of farms continuously dropped from nearly 61,000 in 2008 to approximately 51,000, with an expansion of farm size from 17.4 up to 20.5 ha in 2018 (Bundesamt für Statistik, 2020). The less favoured mountain areas suffer more from a decline in farms (Bundesamt für Landwirtschaft, 2021). Livestock farming is predominantly conducted in mountainous and hilly areas, whereas arable farming is practised in the lowlands (Bundesamt für Statistik, 2023). Family farming is the dominant management form; of roughly 150,000 farm workers, only 35,000 are not family related (Bundesamt für Statistik, 2021a). Accordingly, succession usually happens within the family (Grütter, 2019). Nevertheless, the COVID-19 pandemic and restricted borders in Europe highlighted the dependency on foreign harvest labourers who work under precarious conditions (e.g. long working hours, low wages, little information on social security) and are, therefore, difficult to replace (Schilliger, 2021).

The share of organic farming has grown in recent decades and accounted for 16.5% (Bundesamt für Statistik, 2021a) of agricultural land in 2019. Officially, the minimum requirements for organic production are stipulated by organic regulations. However, the private sector organisation *Bio Suisse* represents the association of Swiss organic agricultural organisations and the national organic brand and, thus, relies on stricter guidelines. Organic produce carrying the *Bio Suisse* label^5^ comprises approximately 60% of the organic market in Switzerland. A farmers’ organisation, together with the retailer *Migros*, developed the Integrated Production (IP) label in the late 1980s as a hybrid between conventional and organic farming that relies on moderate use of chemicals (Belz, 2006). Within the *Swiss Farmers’ Union*, farmers are organised via their cantonal associations. According to their constitution (Schweizer Bauernverband, (n.d.)), their central mission comprises among others the representation of farmers’ interests at the national and international levels to secure their income and livelihood.

#### Policy

Article 104^6^ of the Swiss Constitution regulates agricultural policy and was introduced by referendum in 1996. It provides a legal basis for multifunctional agriculture and ‘can be seen as a new social contract between Swiss farmers and the population, aimed at sustainability’ (Belz, 2006 p. 195). Article 104a^7^ on food security was added in 2017. The Swiss agricultural policy has a budget of CHF 3.7 billion (2021, € 3.8 billion), accounting for approximately 5% of the total Swiss state budget (Schweizerische Eidgenossenschaft, 2021).

Based on direct democracy, a federal popular initiative enables Swiss citizens to develop and articulate proposals to revise the Federal Constitution outside of legislative and executive processes. Important initiatives in 1996, 2005, and 2017 fundamentally changed the agricultural policy by favouring sustainability goals such as multifunctionality, GMO-free agriculture, and food security in the Swiss Constitution. Since 2012, several popular initiatives^8^ related to food and agriculture have been launched but were rejected in the referendum.

Since the 1990s, more than 100 bilateral agreements, which regulate Switzerland’s trade relations with EU countries, have been concluded with the European Union (EU). As the EU is Switzerland’s most important trading partner in the agricultural sector, 77% of imports and 59% of exports were conducted with the region in 2018 (Bundesamt für Landwirtschaft, 2021). Protocol No. 2 of the Swiss EC Free Trade Agreement of 1972 allows Switzerland to levy customs duties on imports of agricultural products available in Switzerland. However, a bilateral agreement on trade in agricultural products in 1999 aimed to eliminate tariff and non-tariff trade barriers. Switzerland has traditionally had a strong, export-orientated manufacturing sector and joined the WTO in 1995 (Weder, 2018). Since 2019, however, export subsidies are no longer WTO compliant and have, therefore, been abolished. To evade WTO regulation, Switzerland invented a measure of compensatory payments to food exporters for expensive raw materials, such as milk and wheat, from protected production directly to the farmers (Economiesuisse, 2019).

#### Trade and retail

The level of self-sufficiency of gross agricultural products is nearly 60% (Bundesamt für Landwirtschaft, 2021); however, its net value (without imported animal feed) is discussed to be much lower (25%, Bosshard, 2009). Imports of energy- or protein-rich concentrated feed accounted for 55% in 2018 (Baur & Krayer, 2021). Exported goods are mainly processed foods (e.g. chocolate, coffee, lemonade) and dairy products (e.g. cheese) (Bundesamt für Landwirtschaft, 2021). Several Swiss communes attract companies with low tax levels; thus, some large, export-orientated transnational corporations in the agro-food business, such as *Nestlé*, *Glencore*, *BASF Schweiz*, *Bayer Schweiz*, *Leu* + *Gygax*, *Omya Schweiz Agro*, *Stähler Suisse*, and *Syngenta Schweiz*, are based in Switzerland.

Regarding retail, the two leading food retailers (*Coop* and *Migros*), which share approximately 80% of all food sales, are Swiss companies organised in the form of cooperatives under market economy principles (Jungmeister, 2020). Thus, they also dominate the organic retail market, with the organic pioneer *Coop* accounting for approximately 41.2% of organic sales in 2021 and *Migros* accounting for 31.2% (Bio Suisse, 2022). Both have introduced their own sustainability labels. In the 1970s, Migros had already introduced integrated production standards with its ‘M-Sano-programme’. *Coop* subsequently introduced the *Naturaplan* (Belz, 1999) with *Bio Suisse* in the early 1990s, stimulating development of the organic farming niche. *Migros* established its label, *Migros Bio*, in the late 1990s, but did not integrate the *Bio Suisse* bud label (Belz, 2006). Additionally, both sell locally produced food under the label ‘Miini region’ (*Coop*) or ‘Aus der Region, Für die Region’ (*Migros*).

The steadily growing (Jungmeister, 2020) upstream agricultural producer, *Fenaco*, dominates feedstuff and fertiliser trading and controls agricultural engineering, seeds, and other domains. Interestingly, *Fenaco* is also cooperatively organised and owns the retailer *Volg* and 183 LANDIs (farming cooperatives) and has more than 43,000 members, including 23,000 farmers.

#### Consumption

In 2019, an average Swiss household spent approximately 12% of its budget (CHF 1200 per month) on nutrition, including meals and drinks in restaurants (Meyre, 2022). The consumption of organic food (including drinks) increased from 7% in 2010 to 12% in 2018 (Bundesamt für Statistik Neuchâtel, 2021b) and even more during the COVID-19 pandemic in 2019 −2020 (Bundesamt für Landwirtschaft, 2022).

Regarding diet, the number of vegetarians and vegans is on the rise (even if the number itself is unclear), along with the population consuming increasing amounts of meat, thus, resulting in a stable per capita meat consumption (Mann & Necula, 2020). Food loss and waste occur along the entire food value chain, causing a 48% leakage of the total calories produced. Households are responsible for almost half of the total avoidable waste (in terms of calorific content) (Beretta et al., 2013). To tackle this problem, which also concerns the 2030 Agenda for Sustainable Development, the Federal Council recently passed an action plan to reduce food waste in trade and consumption by 50% (Schweizerische Eidgenossenschaft, 2022).

#### Alternative food systems

AFS have a long tradition in Switzerland. *Les Jardins de Cocagne*, established in 1978, is one of the first examples of community-supported agriculture (CSA) worldwide (Scherer & Rist, 2017). Approximately 60 CSAs currently provide food to an average of 450 consumers per CSA (Bigler, 2015).

In 2015, the four largest Swiss cities (Zurich, Geneva, Basel, and Lausanne) signed the Milan Urban Food Policy Pact^9^ aimed at developing and integrating an urban food policy. Accordingly, Geneva strives for an integrated approach to govern the food system. The city provides technical support within the food system, such as guides for restaurant owners to source local food (Candel, 2020). However, the frameworks integrating (rural) food production and (urban) food consumption are limited (Moschitz, 2018).

## Results and discussion: framing the food regime in Switzerland

To frame the contemporary food regime in Switzerland, I present the empirical results and discuss them in light of the research objectives. The number in parentheses refers to the respective experts (see Table 1).

### Agency in the food regime in Switzerland

In seeking to comprehend agency within the contemporary food regime, this study examines the collective agency of actor-networks from a relational perspective. Relying on the collected data, experts ascribe collective agency to diverse actors within the contemporary dominant food regime in Switzerland (CFRS) that form actor-networks., e.g. pertinent media and unions involved in farming, processing, and consumption. Nevertheless, the data does not allow for the identification of collective agency among AFS actors, implying a limited capacity on their part to bring about transformative changes in the food regime. Below I will discuss these findings in detail.

#### Collective agency and actor-networks that shape the food regime

The identified multiple actor-networks, characterized by their inherent collective agency, enabling them to significantly influence the CFRS. These actor-networks operate across diverse levels, including the political sphere and media, the retail sector, farm production-related industries, as well as involving consumers and NGOs. The subsequent sections will elaborate on these identified actor-networks.

The politically independent actor-network *Agrarallianz*^10^ represents a broad range of members and diverse interests to ‘support the Federal Office for Agriculture towards the Swiss Farmers’ Union’ (1, see also 12). Contrastingly, the *Swiss Farmers’ Union* (not a member of the *Agrarallianz*) focuses on farmers’ interests and, consequently, opposes some of *Agrarallianz*’s positions. Interestingly, this coalition acts to support the Federal Office for Agriculture in opposition to the *Swiss Farmers’ Union*, as one expert pointed out (3). This need for opposition underscores the collective agency of the *Swiss Farmers’ Union*, representing farmers and, thus, the food from somewhere sub-regime that is still enmeshed with food from nowhere, considering the concentrated feed imports. The *Swiss People’s Party* (Schweizerische Volkspartei, (n.d.)) is a national-conservative, right-wing populist party that represents a more bourgeois, market-conservative, agro-industrial position that is closely related (Schweizer Bauer 2022) to the *Swiss Farmers’ Union* (1, 3). The pertinent media in the agricultural sector are the farmers’ journals *Schweizer Bauer* and *Bauern Zeitung*. Although both influence the opinion-making of the farming community, experts complain that these media only transmit one perspective, especially regarding popular initiatives (3, 12). Furthermore, experts critique the *Swiss Farmers’ Union* because it hinders the practical implementation of transformation and innovation in food systems. Here, it aims to protect the traditional way of farming and refuses change (1, 9, 11, 12) (see also Richter et al., 2023). For example, it impedes the promotion of a vegetarian/vegan diet and is ‘over-critical’ toward plant-based milk substitutes (11, 12), whereas the reduction of meat consumption is seen by experts as one ‘adjusting screw’ towards sustainability in the contemporary food regime (1, 2, 8, 9, 11; see also Mann & Necula, 2020). Here, media, the *Swiss Farmers’ Union* as well as the traditional livestock system build an actor-network that demonstrate strong collective agency that depends on strong mutual relations. The *Swiss Farmers’ Union* is further denounced as hampering the development of an ecological way of farming (e.g. regarding the regulation of pesticides). It demonstrates a defensive attitude rather than tackling the challenge (1), “And that’s a bit symptomatic of the attitude of the Farmers’ Union” (1). Related to this issue, experts (9, 12) find fault with the *Swiss People’s Party,* which hampers a sustainable transformation of food systems. Farming representatives’ forming an actor-network that demonstrate a collective defensive attitude towards the transformation of food systems presented above makes it difficult for single farmers to address critics of meat production and consumption (7).

Interestingly, as a retailer, *Migros* has been making progress in developing alternative meat production, thus contributing to the transformation towards sustainability within the food regime. Therewith, it challenges traditional livestock farming and meat consumption. Even if official state regulations and implementations to address the reduction of meat production and consumption are missing at large, a private economic actor has the capacity to act intentionally and instigate meaningful change within the system, thus demonstrating collective agency within the dominant food regime.

Additionally, the agricultural production upstream, with its monopolistic position (8), demonstrates strong collective agency within the CFRS. It acts as an interactant under the pressure of farmers’ (and the *Swiss Farmers’ Union’s*) demands (3) and, therefore, strives to secure farmers’ income and livelihood to guarantee competitive farming in Switzerland. In the processing industry, several associations are organised under the umbrella of the *Federations of the Swiss Food Industry* that are influential in public debate on popular initiatives (1, 3).

The two main retailers in Switzerland build a robust actor-network and hold strong collective agency in the dominant food regime. With the organic turn of *Coop*, the retailer expanded its role in developing the Bud label, and gained corporate power as a capitalist company that progressed towards ‘green capitalism’ (see Friedmann, 2005, 2016). Simultaneously, the *Bio Suisse Union* that provides the Bio Suisse label not only depends on *Coop* as a client but also owes its origins to the retailer. Today, *Migros* aims to produce and process organic products under the Bio Suisse label. Owing to the *Migros Group*’s size, this massive transformation challenges the *Bio Suisse Union* bureaucratically (9). There is awareness of the duopolistic situation in retailing, but the fact that both are national businesses is regarded positively and, thus, reliability is assumed (7, 9). However, one expert points out their powerful position:

‘And they actually tell you how to do it. Luckily, they’re in Swiss hands, reasonable, let me put it that way,[they] have good ideas, but in the end {uh} there’s no getting around it if one of these retailers says, “My bread grain now has to be produced herbicide-free”, then that’s the way it is’ (7).

Others criticise informal price agreements and the fact that competition law was not applied—‘Yes, if there are only two it’s not that difficult, is it (laughs)’ (3). The retailers’ collective agency is further expressed through private labels, which allow them to influence food production (see also Schermer, 2022); for example, *Bio Suisse* implements organic regulation (Bio Verordnung), allowing soy-based animal feed, whereupon the private (*Migros*) label, *Naturabeef*, interdicts the use of any soy. Additionally, experts criticise the main retailers (4) as they are subject to economic interest (see Clapp & Scrinis, 2017, for criticism). Interestingly, although Swiss retail and upstream corporate powers are legally organised as cooperatives, their ambitions underlie market economy principles (e.g. maximising profits). Thus, their neoliberal actions (e.g. developing a strong segment of their own brands, contracting of anonymous manufacturers (see Burch & Lawrence, 2009)) may be confused with the behaviour of cooperatively organised non-profit organisations (so-called ‘Sozialgenossenschaften’). Nevertheless, cooperatives are regarded positively by the Swiss population (Jungmeister, 2020), which strengthens their collective agency in the CFRS.

Popular initiatives rooted in the direct democratic system ascribes individual agency to the citizens (consumers) to impact the CFRS. Even if most popular initiatives have been rejected, their objectives gained the attention of regime actors and placed values (1), such as equality, biological diversity, health, ecological effects of farming, fair trade, and agricultural labour, on the agenda of several main actors in the CFRS (see Friedmann, 2005). Thus, direct democracy enables consumers’ to take in collective agency in the CFRS.

Regarding positions to challenge the dominant food regime, NGOs, such as *Vision Landwirtschaft*, or producer unions, such as *Uniterre*, demonstrate collective agency to confront the food from nowhere sub-regime directly and claim the integration of the food from here sub-regime. Hence, a bipartition exits among collective actors: they either favour the food from somewhere sub-regime (*Swiss Farmers’ Union*) or the food from here sub-regime (*Uniterre, Vision Landwirtschaft*), with the *Agrarallianz* as an actor-network somewhere in between.

#### Limited collective agency of AFS actors in the food regime

Regarding AFS, experts refer to only a few examples, such as start-ups or CSA. These start-ups work with plant-based meat producers, such as *Planted*(11), that however acts within the industrial production logic. Other private innovative start-up enterprises, such as *kitro* or *foodwards*, aim to tackle food waste (11); however, relevant state regulations must still emerge (see Schweizerische Eidgenossenschaft, 2022).

The missing awareness of AFS actors leads to the conclusion that AFS actors do not build powerful actor-networks, nor do they inherently possess collective agency to significantly influence the CFRS. Whereas the data of the study revealed that within the CFRS, the individual agency (e.g. single consumers, farmers) demonstrates limited impact, the collective form of agency is strongly shaping the contours of the dominant food regime. Here, mutual relations that result in actor-networks ally to achieve strong agency to shape the development of the food regime.

### Interrelationships between the food from nowhere, somewhere, and here sub-regimes

The CFRS is shaped by its heterogeneous structure and the interrelationships of the food from nowhere, somewhere, and here sub-regimes.

The agricultural structure is shaped by the comparatively small average size of family farms that is an expression of peasant farming (van der Ploeg, 2018) in Switzerland. However, these farms depend considerably on cheap external labour for the harvest season. The food that is produced is valued Swiss food from ‘somewhere’ that is culturally rooted and ecologically embedded, but depends on anonymised labour (3), a characteristic of the food from nowhere sub-regime. This situation further reflects the dependency on foreign labour and the undermining of social contexts (Bernstein, 2016) within the local labour market. Considering the financialisation of agriculture mostly reflected in the food from nowhere sub-regime, the pressure for digitalisation among small-scale producers and processors exceeds not only their financial scope (see Prause et al., 2021) but also their capabilities (3).

The 1990s’ agricultural policy reform that implemented a new regulatory framework aligned with WTO directives is a starting point for enabling corporate power (see McMichael, 2009; Smith et al., 2010). This neoliberal ideology favours the internationalisation of national agriculture (Pritchard, 2009), observed in the strong dependency on imports of concentrated feed or raw materials for refining. This development reflects the food from nowhere sub-regime. Contrastingly, the agricultural policy remains protective and Swiss agriculture is not competitive (Belz, 2006), contradictory to the WTO objectives. This scenario has resulted in a bipartition of the contemporary food regime in Switzerland that follows dialectic aims: on the one hand, the globalised strategy enables exports based on raw material imports, as well as the integration of cheap foreign labour, thus sustaining the food from nowhere sub-regime; on the other, the localised strategy protects national agriculture in the food from somewhere sub-regime.

Private sector organisations, such as *Bio Suisse Union*, find it difficult to balance economic growth with the preservation of underlying values according to the principles of IFOAM^11^ (7, 9). The following example illustrates this situation: *Bio Suisse* generally promotes gentle processing:

‘And there we have of course friction’ (9) as this creates leeway for interpretation. The importation of organic wine from Argentina was also discussed, which is acceptable from an ecological perspective (transport in bulk) but was ultimately rejected internally by Bio Suisse. However, as an expert stated, ‘The wine is coming to Switzerland anyway. Simply without the Bud label. And if we also vouch for an import, do we then also want to promote ecological agriculture abroad? (9).

The conventionalisation of organic food enabled by the retailer *Coop* who pushed *Bio Suisse* and, therefore, organic food into the food from somewhere sub-regime (Campbell, 2009), which abandoned the central core values of organic^12^ (see Luttikholt, 2007), for example the respect for seasonal cycles over economies (Blancaneaux, 2022). As a consequence for AFS, organic and vegan foods are no longer a unique characteristic of farmers’ markets or organic grocery stores, which weakens their niche. Currently, alternative food networks and alternative economies approaches, such as CSA or urban food policies that strive for food from here, are present in urban areas. By contrast, few such approaches exist in rural areas. Even if CSA were an important niche addressing 15 − 20% of consumers’ needs, in reality this model is not practical (1) as the percentage is much lower (Bigler, 2015). The lack of small-scale regional infrastructure (9) that disappeared with the rise of the food from nowhere sub-regime is problematic for processed food within alternative food networks and alternative economies. As retailers also strive to implement alternative food networks by selling traditionally produced local food, they convey the image of food from here, while the unique selling point for the traditional alternative food networks, such as farmers’ markets or CSA, has disappeared; accordingly, this situation limits further development of alternative food networks and alternative economies. Nevertheless, national ambitions to strengthen the ‘food from here’ sub-regime can be observed in the new feeding policy for organic farming, which only accounts, however, for a small share of farms in Switzerland.

A demand for environmental standards and new producer–consumer relationships has emerged within the third food regime. This consumer demand is reflected in several popular initiatives, such as ‘Multifunctional Farming’ or ‘Fair Food’, and has been adopted by the two leading retailers, *Coop* and *Migros.* With the implementation of environmental standards in the organic enterprises of *Coop* and *Migros*, the retailers demonstrated the adaptability of capitalist companies towards ‘green capitalism’ (see Friedmann, 2005, 2016). The demand for new producer–consumer relations (see Schermer, 2015) in the third food regime is variously expressed, such as the rise in direct marketing of farm products that skip intermediaries, as well as CSA initiatives that also aim to link remote rural producers and urban consumers within one CSA. On the retail side, these initiatives satisfy consumers’ desires for cultural and biological diversity, fair trade, and social justice (see Friedmann, 2016) by offering locally and traditionally produced food, thus conveying the image of a close producer–consumer relationship. Social relations (see Otero, 2016; Tilzey, 2019) have, therefore, entered the economic stage and these local and traditional foods demonstrate the initial commodification of social relations. Based on what Friedmann (2016) called ‘green capitalism’, the term ‘socially green capitalism’ can be applied when private economic enterprises exploit this demand for social embeddedness for economic purposes. Thus, retailers have already implemented strategies of food from somewhere and now strive to do so with the food from here sub-regime to gain profits (see Rosol and Barbosa 2020).

Simultaneously, with a strong segment of their own brands, retailers contract anonymous manufacturers (see Burch & Lawrence, 2009); accordingly, much of their action lies within the food from nowhere sub-regime. A financialisation of the FR, where private companies in the food sector behave like financial institutions (Burch & Lawrence, 2009), is further observed with the two leading retailers. Their diversification and vertical integration (e.g. the *Migros Bank*) demand massive financial transactions. Additionally, the fact that food commodity companies, based mainly in the Lake Geneva region, engage in food speculation was negatively perceived among the Swiss population. However, the ‘Stop Speculation’ initiative to restrict financial institutions was rejected (see footnote 15).

## Conclusion

This study analyses the dominant food regime in Switzerland by shedding light on its heterogeneous structure and the dynamics of agency within. This conceptual-empirical contribution informs debates on food regime theory and its empirical investigation that considers agency and AFS within the corporately governed food regime. Given that the presented study relies on an explorative, qualitative approach to social research, reconstructing selected perspectives on the food regime in Switzerland, a more expansive empirical approach is warranted for a comprehensive understanding.

This study highlights diverse manifestations of collective agency within the CFRS, involving private companies; pertinent media; associations; and unions across farming, processing, and consumption sectors. Consequently, the dynamics of the food regime are shaped by contested social practices, which are influenced and interpreted through social agency, as highlighted in Atkins and Bowler (2003). Corporate power is present in the CFRS, which valorises the demand for sustainability beyond the ecological realm for social relations’ own economic purposes, thus leading to the phenomenon of ‘socially green capitalism’. Moreover, corporations as an actor-network hold strong collective agency. Therewith, they are shaping social regulations that leave behind small-scale actors—producers as processors—in the FR. Within the CFRS, collective agency is among the few powerful actors that promote the different sub-regimes of food from nowhere and somewhere, and to a lesser extent with representatives from the food from here sub-regime. The results underscore the power that corporations wield and the strategies of adopting AFS approaches. They echo Lawrence and Burch’s (2009) findings that the power shift to retailers in the food system has not changed power relations for the better. Here, Swiss policy and consumers seek FR transformation (e.g. decrease of food waste, reduction of meat consumption); however, influential organisations hamper this change as they stick to traditional ways of farming (see Richter et al., 2023). It is a sort of cultural resistance that represents the fear of losing one’s identity and livelihood.

The national expression of the CFRS corresponds to global trends, but it also depends to some extent on regional and national interpretations of the food regime setting, which exhibit significant variations. These results echo Smith et al. (2010) who observed that divergent demands shape the corporate-environmental food regime that has led to strong counter-movements. Here, the food from nowhere sub-regime, which is based on a strong export orientation and is highly dependent on food imports resulting in the need for free trade, is in persistent conflict with the food from somewhere or even food from here sub-regimes that strongly protect national agriculture and highly value ‘Swiss’ food. Over 10 years ago, Campbell (2009, p. 318) highlighted ‘food from somewhere’ ‘as a small but important new set of counterlogics’; today, it has been pushed forward, completely adopted, not to say exploited, by dominant retailers in Switzerland. Moreover, they even strive to adopt food from here strategies, whereas there are still substantial barriers to break through (e.g. the underlying values of alternative food networks and alternative economies). Overall, this adoption limits the growth of alternative food networks and alternative economies to overcome their existence in a niche.

Whereas debates on food regimes are often dichotomised into conventional or alternative agriculture, this empirical study demonstrated the overlap and interdependencies of such sub-regimes that might reflect an unstable period of transition. To solve the current impasse of food regime theory, it is however necessary to find consent about an emerging, existing, or ending third food regime (for detailed discussion on this, see Howard, 2021).

## Data Availability

The data that support the findings of this study are available from the corresponding author upon reasonable request.

## Footnotes

1 For a detailed discourse on the distinction, see Bernstein (2016).

2 Alternative food systems refer to alternatives to conventional or industrial food systems in response to environmental, health, justice, and ethical concerns (Rosol 2020, p. 56).

3 Prepared in the realm of the project ‘Exploring values-based modes of production and consumption in the corporate food regime’, where Switzerland represents one case study.

4 In parallel with the two other case study countries Czech Republic and Argentina.

5 Bio Suisse, established in 1981, stands as the foremost organic organization in Switzerland and holds ownership of the Bud trademark. The association’s logo, featuring the Bud with the Swiss cross alongside the Bio Suisse emblem, serves as both the organizational insignia and the distinctive mark for its member farms.

6 Art. 104: The Confederation shall ensure that the agricultural sector, by means of a sustainable and market-oriented production policy, makes an essential contribution towards:

- the reliable provision of the population with foodstuffs;
- the conservation of natural resources and the upkeep of the countryside;
- decentralised population settlement of the country.

7 Art. 104 a: To guarantee the supply of food to the population, the Confederation shall create the conditions required for:

- safeguarding the basis for agricultural production and agricultural land in particular;
- food production that is adapted to local conditions and which uses natural resources efficiently;
- an agriculture and food sector that responds to market requirements;
- cross-border trade relations that contribute to the sustainable development of the agriculture and food sector;
- using food in a way that conserves natural resources.

8 The ‘Stop Speculation’ initiative in 2016 sought to restrict financial speculation on food and agricultural commodities (Vuilleumier 2017). The ‘Fair Food’ initiative, to improve quality standards for imported food, was rejected in 2018. In 2021, two more initiatives related to agriculture and food were abandoned: the ‘Pesticide Initiative’ proposed to ban all synthetic pesticides from agricultural and non-agricultural uses, such as food processing and landscape management, and to prohibit all imports of food produced with synthetic pesticides. Furthermore, the ‘Drinking Water Initiative’ envisaged tightening the entry criteria for direct payments (cross compliance requirements) (Finger 2021). A referendum rejected in 2022 aimed to abolish mass livestock farming by incorporating the dignity of farm animals into the constitution.

9 This is an international agreement among cities worldwide, committed to developing sustainable food systems that are inclusive, resilient, safe, and diverse, and providing healthy and affordable food to all people in a human rights-based framework, minimising waste and conserving biodiversity while adapting to and mitigating impacts of climate change (www.milanurbanfoodpolicypact.org/).

10 *Agrarallianz* unites organisations representing consumers, the environment, animal welfare, and agriculture (such as Bio-Suisse, IP Suisse, Demeter, FiBL, Slow Food Switzerland, and Vision Landwirtschaft) to favour the dialogue between ‘hay fork and dinner fork’ by considering the entire value chain.

11 The four principles of organic agriculture as defined by the International Federation of Organic Agriculture Movements (IFOAM) are health, ecology, fairness, and care (Luttikholt 2007).

12 The values of organic agriculture, according the International Federation of Organic Agriculture Movements (IFOAM), go beyond simple technical aspects of farming and are based on four main principles: principle of health: organic agriculture should sustain and enhance the health of soil, plant, animal, human and planet as one and indivisible; principle of ecology: organic agriculture should be based on living ecological systems and cycles, work with them, emulate them and help sustain them; principle of fairness: organic agriculture should build on relationships that ensure fairness with regard to the common environment and life opportunities; principle of care: organic agriculture should be managed in a precautionary and responsible manner to protect the health and well-being of current and future generations and the environment.

## Code availability

Application of MaxQDA.
